# Endochitinase and Chitobiosidase Production by Marine *Aeromonas caviae* CHZ306: Establishment of Nitrogen Supplementation

**DOI:** 10.3390/bioengineering10040431

**Published:** 2023-03-29

**Authors:** Flavio Cardozo, Valker Feitosa, Omar Pillaca-Pullo, Adalberto Pessoa

**Affiliations:** 1 Departamento de Microbiologia, Instituto de Ciências Biomédicas, Universidade de São Paulo (USP), São Paulo 05508-000, Brazil; 2 Departamento de Tecnologia Bioquímico-Farmacêutica, Universidade de São Paulo (USP), São Paulo 05508-000, Brazil; 3 Departamento de Medicina e Enfermagem, Universidade Federal de Viçosa (UFV), Viçosa 36570-900, Brazil; 4 Centro de Investigación en Biodiversidad para la Salud, Universidad Privada Norbert Wiener, Lima 15046, Peru

**Keywords:** endochitinase, chitobiosidase, *Aeromonas caviae*, nitrogen sources

## Abstract

*Aeromonas caviae* CHZ306, a marine-derived bacterium isolated from zooplankton, can use chitin (a polymer of a β-(1,4)-linked *N*-acetyl-D-glucosamine) as a carbon source. The chitin is hydrolyzed by chitinolytic enzymes, namely endochitinases and exochitinases (chitobiosidase and *N*-acetyl-glucosaminidase). Indeed, the chitinolytic pathway is initiated by the coexpression of the enzymes endochitinase (EnCh) and chitobiosidase (ChB); however, few studies, including biotechnological production of these enzymes, have been reported, although chitosaccharide are helpful in several industries, such as cosmetics. This study demonstrates the potential to maximize the simultaneous EnCh and ChB production by nitrogen supplementation on culture media. Twelve different nitrogen supplementation sources (inorganic and organic) previously analyzed in elemental composition (carbon and nitrogen) were tested and evaluated in the Erlenmeyer flask culture of *A. caviae* CHZ306 for EnCh and ChB expression. None of the nutrients inhibited bacterial growth, and the maximum activity in both EnCh and ChB was observed at 12 h, using corn-steep solids and peptone A. Corn-steep solids and peptone A were then combined at three ratios (1:1, 1:2, and 2:1) to maximize the production. The high activities for EnCh (30.1 U.L^−1^) and ChB (21.3 U.L^−1^) were obtained with 2:1 corn-steep solids and peptone A, corresponding to more than 5- and 3-fold enhancement, respectively, compared to the control condition.

## 1. Introduction

Chitin, the second most abundant natural polymer, is a nitrogen-containing polysaccharide composed of a β-(1,4)-linked *N*-acetyl-D-glucosamine (GlcNac) [1,2]. Chitin is widely distributed as a structural component of crustaceans, insects, and other arthropods and as a component of most fungi and some algae cell walls. Approximately 75% of the total weight of shellfish, such as shrimp, crabs, and krill, is considered waste, and chitin comprises 20–58% of the dry weight of the said waste [3]. Annually, more than 1000 metric tons of chitin are synthesized in the aquatic biosphere and deposited in marine sediment; thus, chitinolytic bacteria efficiently degrade this material to maintain the balance in the marine ecosystem [1,4]. Bacteria can synthesize chitinases to hydrolyze chitin as a carbon, nitrogen, and energy source, consequently making possible the recycling of chitin in the environment [2,5]. The chitinases (EC 3.2.11.14) are an efficient group of multiple enzymes classified into glycoside hydrolase families [6,7]. The chitin degradation by chitinases proceeds via well-characterized consecutive steps; these enzymes are classified into two main categories: endochitinases—EnCh (EC 3.2.1.14), which produce small oligosaccharides and exochitinases—ExCh (EC 3.2.1.29), which are further subdivided into two subcategories: (i): chitobiosidase—ChB (EC 3.2.1.29), which catalyzes the release of *N,N*’-diacetylchitobiose [(GlcNAc)_2_], and (ii): *N*-acetyl-D-glucosaminidase—NAG (EC 3.2.1.30), which cleaves the previous oligomeric products into monomers of GlcNAc [1,4,7]. In bacteria, chitinases play a role in nutrition and parasitism [8]. In contrast, chitinases have been used for chitosaccharide production (e.g., oligomers and monomers of GlcNAc) in food, cosmetic, pharmaceutical, and biomaterial fields [1,9]. Actually, (GlcNAc)_2_ (*N*,*N*’-diacetyl-chitobiose) has significant relevance to the biotechnological, pharmaceutical, cosmetic, food, and agriculture industries due to its wide range of biological activities [7,10]. In this sense, (GlcNAc)_2_ can be used as a cosmetic ingredient due to its high solubility and as a building block for polysaccharide synthesis [7,11]. From an economic perspective, the market price of (GlcNAc)_2_ is approximately US $2000 per g, thus representing about 10,000 times more than GlcNAc [12].

Heterotrophic bacteria from aquatic environments related to chitin decomposition include the genera *Aeromonas, Enterobacter, Chromobacterium, Arthrobacter, Flavobacterium, Serratia, Bacillus, Erwinia,* and *Vibrio* [13]. Members of the *Aeromonas* species are widely distributed in nature, and strains isolated from soil and seawater are capable of secreting chitinases [2,4,6]. *Aeromonas* species, including 36 members, are described as Gram-negative, motile, facultatively anaerobic, rod-shaped, and oxidase-positive bacteria [14,15]. However, some *Aeromonas* species have been reported to induce pathogenicity (i.e., *A. hydrophila, A. caviae,* and *A. veronii* producing systemic infections in humans) [16]. The pathogenic potential of *A. caviae* does not represent an obstacle to applying their crude enzyme extracts to GlcNAc production since they are just intermediaries in the process [9]. Thus, *Aeromonas* members have been attractive novel candidates for technological areas such as biocatalysts, bioremediation, and syntesis of polyester [17] and chitin oligosaccharide production [5,9,18,19].

On the other hand, nitrogen is an essential nutrient for all life forms and is used to synthesize many macromolecules such as proteins, nucleic acids, lipids, and carbohydrates [20,21,22]. This element supports growth rate, and yield can play a vital role in the economic efficiency of the bioprocess [23,24,25]. Still, nitrogen limitation causes growth retardation due to the expansion of the G1 phase of the cell cycle through a reduction in ribosomal biogenesis and translation [26]. Therefore, obtaining nitrogen from the external environment is essential for all living organisms [27]. Assimilable inorganic nitrogen sources include nitrates, ammonia, and ammonium salts. Organic nitrogen is mainly obtained from urea and complex sources such as yeast extract, meat extract, tryptone or peptone, and whey, providing growth factors [28,29,30]. Therefore, culture media optimization is critical to maximizing yield and productivity, [3]. Although some microorganisms can use around 30 distinct nitrogen-containing compounds [21], previous studies included the evaluation of eleven sources for *Aeromonas* sp. [31] but not for specific *A. caviae*; therefore, screening twelve nitrogen sources, including elemental composition, in this bacteria is the goal of our work. In the current study, we demonstrate the influence of nitrogen supplementation on culture media for chitinase production using marine-derived *A. caviae* CHZ306. For this, we evaluated the EnCh and ChB production in culture media supplemented with different inorganic and organic nitrogen sources to maximize the production of chitinases in industrial bioprocesses.

## 2. Materials and Methods

### 2.1. Microorganisms and Growth Conditions

*Aeromonas caviae* CHZ306, a marine chitinolytic bacterium isolated from zooplankton samples from the coast of São Paulo state, Brazil (23°59′13” S, 46°22′26” W) [32], was investigated in this study. *Aeromonas caviae* CHZ306 was characterized at the genus and species levels by complete 16S rRNA gene sequencing and multilocus sequence analysis (MLSA) [9]. To better understand the chitinase diversity and select specific enzymes for chitin derivative production, the genome of *Aeromonas caviae* CHZ306 was sequenced, and its draft genome sequence was deposited at DDBJ/ENA/GenBank under the accession number MDSC01000000 [33]. The strain was grown on colloidal α-chitin agar plates at 28 °C for 96 h, according to the method described by Souza et al. (2009) [32]. To prepare the pre-inoculums, approximately five colonies were added to a 250 mL Erlenmeyer flask containing 100 mL of sterile colloidal chitin broth and incubated in an orbital incubator at 180 rpm and 28 °C for 24 h to obtain a cellular concentration of approximately 5.0 × 10^5^ CFU.mL^−1^.

### 2.2. Evaluation of Nitrogen Supplementation in Bacterial Growth and Chitinase Production

The ability of *A. caviae* CHZ306 to utilize inorganic and organic compounds as a supplementary nitrogen source was tested using the mineral-salt medium described below, in which either inorganic or organic compounds were present at a concentration of 0.2 g.L^−1^. Colloid α-chitin (10 g.L^−1^) was used as the substrate. The broth medium base was composed of minerals and salts prepared to 1 L with: 0.2 g KH_2_PO_4_, 1.6 g K_2_HPO_4_, 0.2 g MgSO_4_.7H_2_O, 0.1 g NaCl, 0.01 g FeSO_4_.7H_2_O, and 0.02 g CaCl_2_.2H_2_O [32]. This mineral-based broth media was supplemented with 10 g.L^−1^ colloidal α-chitin and twelve different nitrogen sources listed in Table 1. The final pH medium was adjusted to 7.0. The cultures were carried out using a 250 mL Erlenmeyer flask containing 100 mL of medium with 1 mL pre-inoculum grown. The cultures were placed in an orbital incubator at 180 rpm, 28 °C, and samples (500 μL) were periodically collected for up to 96 h and evaluated for cell growth and chitinase activity.

### 2.3. Improvement of Endochitinase and Chitobiosidase Production by Nitrogen Source Mixture

The mineral-based medium described in the previous item included 10 g.L^−1^ of colloidal α-chitin, which was supplemented with mixtures of corn-steep solids and peptone A in 1:1, 1:2, and 2:1 proportions to reach a supplementation of 0.2 g.L^−1^ nitrogen (specific quantities are shown in Table 2). The cultures’ conditions and analysis were carried out according to item 2.2.

### 2.4. Analytical Methods

#### 2.4.1. Elemental Composition

Initially, the colloid chitin and complex nitrogenous sources were digested with concentrated HNO_3_ and H_2_O_2_ (30% *w*/*w*) with heating at 925 °C. Elemental composition for carbon (C) and nitrogen (N) was determined using an Optical Atomic Emission Spectrometer with Inductively Coupled Plasma (ICP-OES, Radial) model Arcos-Spectro^®^ (Ametek, Berwyn, PA, USA). Meanwhile, the C and N contents of chemically defined nutrients were calculated based on chemical composition and molecular mass.

#### 2.4.2. Bacterial Growth

The drop plate method determined the cellular growth, expressed by colony-forming units per liter (CFU.L^−^) [34]. Serially diluted samples (10 μL) were plated (in triplicate) on colloidal α-chitin agar plates at 34 °C for 96 h.

#### 2.4.3. Chitinase Activity

Culture samples were centrifuged to collect the supernatant at 3000× *g* for 5 min at 4 °C. EnCh and ChB activities from the supernatant were determined by using the Chitinase Assay Kit (Sigma-Aldrich, Saint Louis, MO, USA), using 10 μL of supernatant and 90 μL of a specific substrate solution (1 mg.mL^−1^ of p-nitrophenol-triacylchitotriose [pNP-(GlcNAc)_3_] or p-nitrophenol-diacetylchitobiose [pNP-(GlcNAc)_2_]). The mixtures were incubated at 37 °C for 30 min; optical density was read at 405 nm using a microplate reader, model Spectramax^®^ plus 384 (Molecular Devices, Sunnyvale, CA, USA). The substrates [pNP-(GlcNAc)_3_] and [pNP-(GlcNAc)_2_] were used as substrates for EnCh and ChB, respectively. One unit (U) of EnCh and ChB was defined as the amount of each enzyme required to release 1.0 μmol of p-nitrophenol (pNP) from each substrate per minute.

### 2.5. Statistical Analysis

Experimental data for bacterial growth and enzymatic activity were presented as the average of three biological assays and expressed as the mean ± standard deviation (sd). Statistically significant differences (*p* < 0.05, 5% significance level) were determined by one-way analysis of variance (ANOVA) followed by Tukey’s multiple comparison post-test using the GraphPad Prism^®^ 7 software.

## 3. Results

### 3.1. Analysis of the Elemental Composition of Chitin and Nitrogenous Sources

Firstly, we determined the C and N content (%) in colloid α-chitin and twelve nitrogen sources, namely ammonium sulfate (M1), ammonium acetate (M2), ammonium nitrate (M3), urea (M4), casaminoacids (M5), meat extract (M6), yeast extract (M7), peptone Bacto (M8), peptone A (M9), peptone G (M10), corn-steep solids (M11), and tryptone (M12), presented in Table 1. The colloid α-chitin contained 44% carbon and 6% nitrogen. Among the chemically defined nutrients (viz. M1–M4), urea (CH_4_N_2_O) had the highest nitrogen content (46.6%). Only ammonium sulfate (NH_4_)_2_SO_4_ (M1) and ammonium nitrate NH_4_NO_3_; (M3) do not have carbon in their chemical composition. Regarding complex nutrients (viz. M5–M12), all of them simultaneously provided carbon (from 33.3% to 44.3%) and nitrogen (from 7.5 to 16%). Then, we estimated the required amount of each nutrient to obtain ~0.21 g.L^−1^ nitrogen in the medium, as proposed by Cardozo et al. [18]. Finally, all these media were tested for bacterial growth and chitinase production.

### 3.2. Evaluation of Nitrogen Supplementation in Bacterial Growth and Chitinase Production

**Figure 1:**
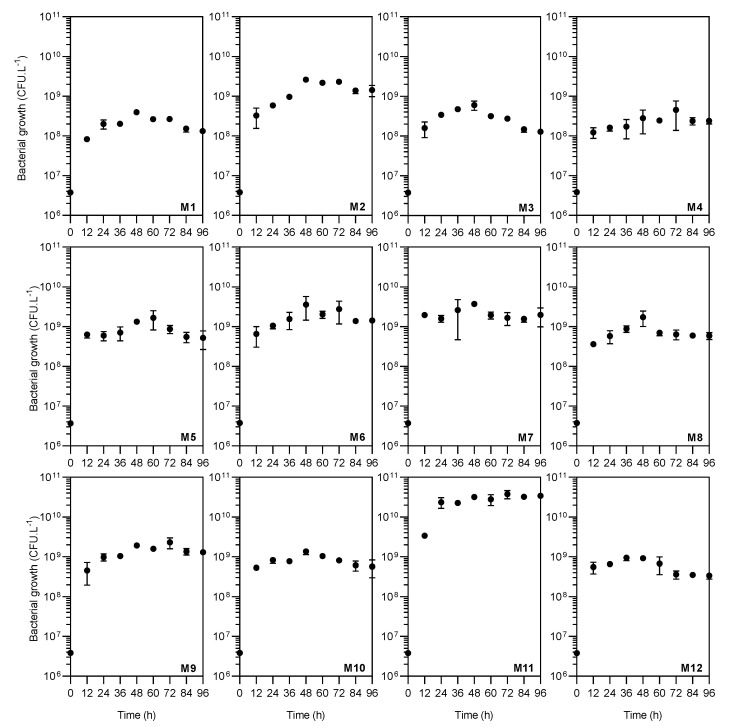
Growth kinetic profiles of *Aeromonas caviae* CHZ306 in the chitin-containing medium under different nitrogen supplementations (viz. M1–M12). M1 (ammonium sulfate), M2 (ammonium acetate), M3 (ammonium nitrate), M4 (urea), M5 (casaminoacids), M6 (meat extract), M7 (yeast extract), M8 (peptone Bacto), M9 (peptone A), M10 (peptone G), M11 (corn-steep solids), and M12 (tryptone). All data represent the mean ± standard deviation (sd).

**Figure 2:**
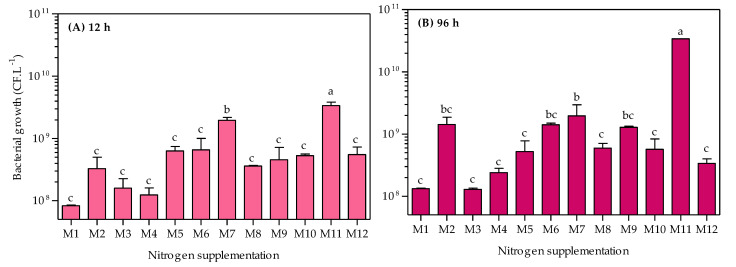
Comparative effect of nitrogen supplementation (viz. M1–M12) on the growth of *Aeromonas caviae* CHZ306. M1 (ammonium sulfate), M2 (ammonium acetate), M3 (ammonium nitrate), M4 (urea), M5 (casaminoacids), M6 (meat extract), M7 (yeast extract), M8 (peptone Bacto), M9 (peptone A), M10 (peptone G), M11 (corn-steep solids), and M12 (tryptone). All data represent the mean ± standard deviation (sd). Different letters indicate statistical differences among columns at the same time (*p* < 0.05).

*A. caviae* CHZ306 cultures were grown in an orbital shaker at 28 °C for 96 h to evaluate inorganic and organic nutrients that could potentially serve as growth substrates. A total of twelve nutrients were tested as nitrogen supplementation (Table 1). As shown in Figure 1, none of the nutrients inhibited *A. caviae* CHZ306 growth, as the bacteria proliferated in all media tested, either with nitrogen supplementation from chemically defined (viz. M1–M4) or complex (viz. M5–M12) sources. Among all the media tested, corn-steep (M11) was far more favorable for bacterial growth and survival. This positive effect of corn-steep solids on bacterial growth was observed early, in the initial 12 h (3.37 × 10^9^ CFU.L^−1^), remaining superior to all other media up to 96 h (3.4 × 10^10^ CFU.L^−1^), as shown in Figure 2A,B. At 96 h, Figure 2B, corn-steep solids (M11) were the only source that achieved ~10^10^ CFU.L^−1^ growth, followed by M2 (ammonium acetate), M6 (meat extract), M7 (yeast extract), and M9 (peptone A), achieving ~10^9^ CFU.L^−1^. Thus, comparing only the chemically defined media (M1 to M4), ammonium acetate supplementation (M2) enabled more remarkable microbial growth (1.42 × 10^9^ CFU.L^−1^), presenting a similar profile of complex sources (viz. M7, M6, and M9). Besides, all nutrients tested, except ammonium nitrate (M3), provide superior bacterial growth than ammonium sulfate (M1, 1.32 × 10^8^ CFU.L^−1^), commonly used in *A. caviae* cultivation [9,34,35]. The data obtained show that *A. caviae* CHZ306 utilized a variety of inorganic and organic compounds, particularly corn-steep solids (2.8 g.L^−1^), as a supplementary source of nitrogen, carbon, and energy for its growth.

**Figure 3:**
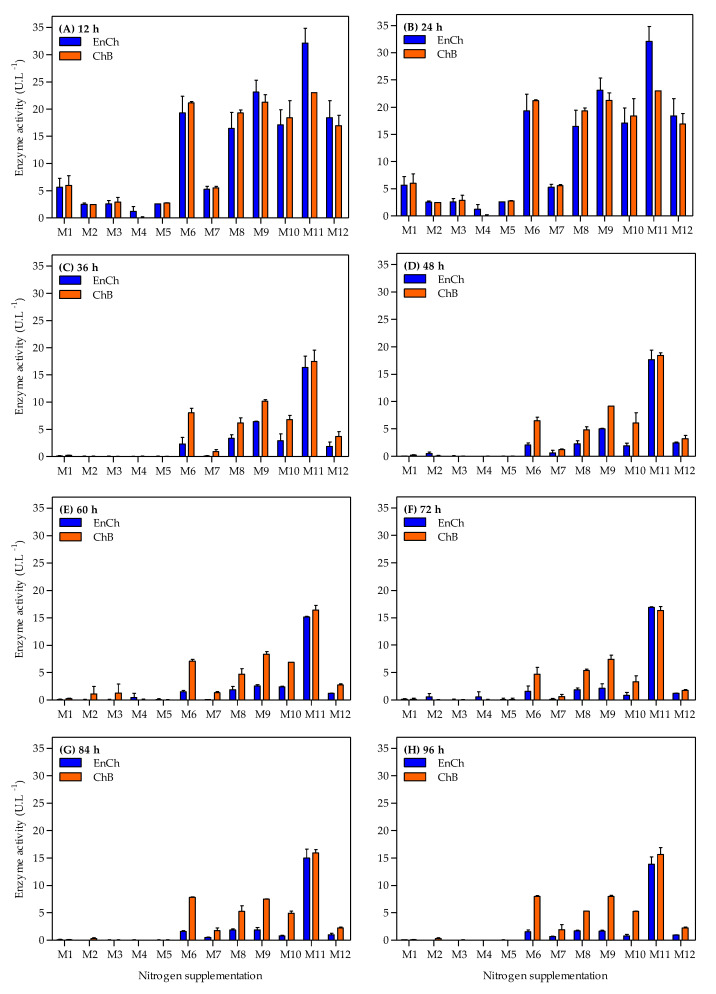
Time-course profiles of endochitinase (EnCh) and chitobiosidase (ChB) activity during *Aeromonas caviae* CHZ306 cultivation in the chitin-containing medium under different nitrogen supplementations (viz. M1–M12). M1 (ammonium sulfate), M2 (ammonium acetate), M3 (ammonium nitrate), M4 (urea), M5 (casaminoacids), M6 (meat extract), M7 (yeast extract), M8 (peptone Bacto), M9 (peptone A), M10 (peptone G), M11 (corn-steep solids), and M12 (tryptone). All data represent the mean ± standard deviation (sd).

**Figure 4:**
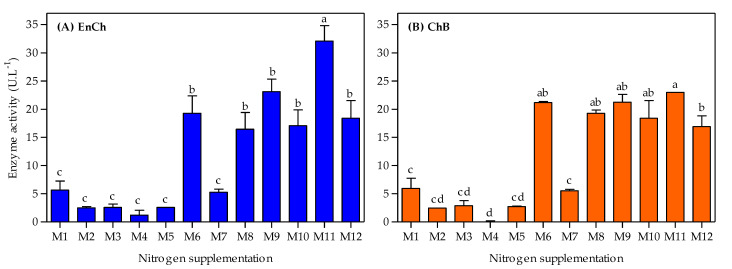
Endochitinase (EnCh) (**A**) and chitobiosidase (ChB) (**B**) activity at 12 h *Aeromonas caviae* CHZ306 is cultivated in the chitin-containing medium under different nitrogen supplementations (viz. M1–M12). M1 (ammonium sulfate), M2 (ammonium acetate), M3 (ammonium nitrate), M4 (urea), M5 (casaminoacids), M6 (meat extract), M7 (yeast extract), M8 (peptone Bacto), M9 (peptone A), M10 (peptone G), M11 (corn-steep solids), and M12 (tryptone). All data represent the mean ± standard deviation (sd). Different letters indicate statistical differences among columns for each enzyme (*p* < 0.05).

Simultaneously, the ability of the *A. caviae* CHZ306 strain to produce both EnCh and ChB enzymes in a chitin-containing medium supplemented with nitrogen sources was tested throughout the 96 h cultivation. The effects of nitrogen supplementation on chitinase production are shown in Figure 3. Overall, the maximum activity in both enzymes was observed at 12 h (Figure 3A and Figure 4), then activity gradually decreased in all tested media (Figure 3B–H). The medium supplemented with ammonium sulfate (M1), previously used in our studies, showed maximum activity of 5.6 and 5.9 U.L^−1^ at 12 h for EnCh (Figure 4A) and ChB (Figure 4B) enzymes, respectively. The highest activity for EnCh and ChB at 12 h was obtained using peptone A-M9 (23.1 and 21.2 U.L^−1^, respectively) and corn-steep solids-M11 (32.1 and 23.0 U.L^−1^, respectively), followed by M6 (meat extract), M8 (peptone Bacto), M10 (peptone G), and M12 (tryptone) that showed similar enzymatic activity. Compared to the ammonium sulfate (M1), the basal medium, peptone A (M9), and corn-steep solids (M11) showed 4.1- and 5.8-fold enhancements in EnCh production and 3.6- and 3.8-fold in ChB production, respectively. Notably, although M2 (ammonium acetate) and M7 (yeast extract) promoted bacterial growth, they were inefficient for EnCh and ChB production. In contrast, *A. caviae* CHZ306 grew less on M8, M10, and M12 media; however, it could express EnCh and ChB on these media.

### 3.3. Improvement of Selected Nitrogen Sources in Endochitinase and Chitobiosidase Production

Table 2 presents three different mixtures of corn-steep solids (M11), and peptone A (M9) formulated to verify the interactions between these two selected nitrogen sources on *A. caviae* CHZ306 growth and chitinase production during 96 h, keeping constant the initial nitrogen supplementation of ~0.2 g.L^−1^.

**Figure 5:**
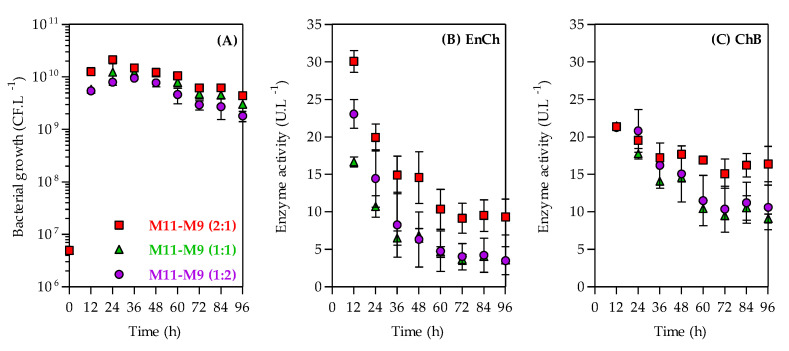
Time-course kinetics profiles of bacterial growth (**A**), endochitinase (EnCh) activity (**B**), and chitobiosidase (ChB) activity (**C**) of *Aeromonas caviae* CHZ306 in the chitin-containing medium under corn-steep solids (M11) and peptone A (M9) mixtures at ratios of 1:1 (green triangles), 1:2 (purple circles), and 2:1 (red squares). All data represent the mean ± standard deviation (sd).

As shown in Figure 5A, the *A. caviae* CHZ306 grew similarly in the three mixtures of corn-steep solids (M11), and peptone A (M9) studied, achieving ~10^9^ CFU.L^−1^ after 96 h of cultivation. Similar to the previous assay, the maximum activity for both enzymes was observed at 12 h, and their activities gradually reduced; notably, the EnCh activity decreased more than ChB over 96 h of cultivation. The EnCh production (Figure 5B) was favored in mediums M11–M9 (2:1) with maximum activity at 12 h (30 U.L^−1^) followed by 23 U.L^−1^ in M11–M9 (1:2) and 16.6 U.L^−1^ in M11–M9 (1:1). Compared to the ammonium sulfate (M1), the control medium, M11–M9 (2:1), M11–M9 (1:2), and M11–M9 (1:1) showed 5.3-, 4.1-, and 2.9-fold enhancements in EnCh production, respectively. The ChB production (Figure 5C) was similar in the three mixtures, also recording a maximum activity at 12 h of 21.4 U.L^−1^ for M11–M9 at a ratio of 2:1, 21.2 U.L^−1^ for M11–M9 (1:2), and 21.5 U.L^−1^ for M11–M9 (1:1). Compared to the control medium (M1), these media showed 3.6-fold enhancement in EnCh production.

## 4. Discussion

In this study, we determined the nitrogenous compounds as potential substrates for the growth of *A. caviae* CHZ 306, particularly in a chitin-containing mineral-based medium. A total of twelve different inorganic and organic nutrients, representing chemically defined and complex sources, serve as substrates for bacterial growth. In general, the composition of the culture medium (including nitrogen sources) significantly influences on cell growth, physiology, and biochemical pathways of microorganisms [36,37]. Thus, the enzyme production varies depending on the nitrogen source; in some cases, there may be a preference for a particular type: nitrogen-rich sources are more rapidly assimilated than nitrogen-poor sources due to the efficiency of transport systems [24,29,38]. In addition, the microorganisms can extract nitrogen from a broad spectrum of organic and inorganic substrates [27]. Therefore, screening and selecting the most suitable nitrogen source for cultivation is essential to maximize product formation.

Ammonium sulfate (M1) and ammonium nitrate (M3) repressed the growth and chitinase production, although ammonium ions could be directly assimilated and act as donors of amino acid synthesis [39]. In M1, the decrease in biomass production is commonly caused by acidification by anion sulfate in media culture [40]. However, ammonium sulfate was the most favorable nitrogen source to produce chitinase by *Aeromonas* sp. JK1 [31], and some authors also reported that *Aeromonas* species could degrade nitrates to nitrites [14]. Moreover, the efficient bacterial growth reached using ammonium acetate (M2) is possible since the salt dissolution in pure water produces a neutral pH solution due to the acidity of NH_4_^+^ (pK_a_ 9.25), which exactly balances the basicity of acetate (pK_b_ 9.25) [41]. In general, inorganic nitrogen sources have an inefficient impact compared to organic sources [42]. However, urea (M4) presents low chitinase production even though the uptake is not proton-coupled and does not cause medium acidification in contrast to ammonium salts [19,40]. However, other *Aeromonas* species, such as *A. hydrophila,* can assimilate significant amounts of urea and nitrogen from ammonium through oxidation by glutamate synthase, while nitrate is utilized through nitrate reductase [43].

In the case of complex nutrients (viz. M5–M12), they could be assimilated by two independent pathways, such as a deamination reaction releasing ammonia or a transamination reaction, where the nitrogen is transferred to the acceptor substrate (α-ketoacid) [27]. Casaminoacid (M5) and yeast extract (M7) had a repressive effect on chitinase production. M5 is an acid hydrolysate of casein containing amino acids and short peptides, although it is adequate for media culture with minimal nitrogen requirements [42,43]. While M7 contains essential nutrients to satisfy nutritional requirements (i.e., carbohydrates, proteins, amino acids, vitamins, and trace elements) [44,45,46], some reports indicated no effect on chitinase production by *A. hydrophila* HS4 [3] and *Aeromonas* sp. JK1 [31]. M7 showed high cell growth and is considered, along with M11, the best source of organic nitrogen for biomass production. However, it is worth mentioning that the metabolic pathways of enzyme synthesis are much slower than the biomass production routes. [20,36]. On the other hand, meat extract (M6), peptone Bacto (M8), peptone G (M10), and tryptone (M12) showed significant chitinase production and growth at 12 h of culture. These results are possible due to composition; for example, M7 is a mixture of peptides and amino acids, nucleotide fractions, organic acids, minerals, and some vitamins [44,47,48]; M8 contains essential amino acids, low molecular peptides, trace elements, and vitamins [44,49]; M10, a pancreatic digest of gelatin, presents higher proline residues [44,50]; and M11, a pancreatic digest (i.e., trypsin hydrolysate) of casein, contains oligopeptides in different lengths and smaller amounts of amino acids and carbohydrates [44,45].

In our findings, peptone A (M9) and corn-steep solids (M11) were the most favorable nitrogen sources for simultaneous EnCh and ChB production, probably because these nutrients provide elements necessary for synthesizing chitinase enzymes. In this sense, both nitrogen sources were selected to continue evaluating chitinase production. M9 is a pancreatic digest of animal tissue and contains a mixture of small and large peptide sizes [44,49,50]. Thus, the nutritional support of peptones and derivates highly depends on the specific peptide (i.e., length and sequence) and free amino acid composition [20]. Moreover, M11 is a soluble solids by-product of the corn milling industry used for microbial growth, providing amino acids, vitamins, minerals, and growth factors [51,52]. The presence of corn-steep solids was also essential to increase the production of the L-asparaginase enzyme by *Leucosporidium scottii* L115 [37].

Comparative data from chitinase production by *Aeromonas* species are presented in Table 3. Some authors indicated that culture medium containing complex nutrients, e.g., peptones, tryptone, yeast extract, and malt extract, despite unknown composition, are cost-effective for processes due to their ability to support maximum product yield and performance, including for large-scale fermentations [36,49].

It is known that the composition of the culture medium affects the final cost of a bioprocess, directly affectingmicrobial growth and product formation [37]. The organic nitrogen from complex sources could be metabolized by cells directly, promoting chitinase production due to its high concentration of most amino acids and growth factors [53]. In this sense, manipulating medium components and concentrations is essential in microbial development because the range of nutrients required depends upon their metabolism [51,54]. Most microorganisms can sense qualitative and quantitative nutrient changes, improving their utilization in competitive environments [21].

Here, maximum chitinase (EnCh and ChB) yields were obtained in the mixture medium M11–M9 (2:1). Indeed, the enzyme production using the mixture of nitrogen sources could be higher than a single nitrogen source [36]. Furthermore, because some organic nitrogen sources are expensive in industrial bioprocess, medium costs can be reduced by replacing them entirely or partially with a less expensive component [50,51]. Medium M11–M9 (2:1) also presents a high C:N ratio, although Arous et al. (2016) [55] indicated that high C:N ratios are favorable for the accumulation of carbohydrates and intracellular lipids instead of enzyme production. This result might be the consequence of channeling more carbon for cell growth and chitinase production.

Moreover, it is suggested that enzyme production was obtained by using quickly metabolized nitrogen sources. Although no significant differences were observed in bacterial growth and chitinolytic activity between medium M11 and the mixture M11–M9 (2:1), the availability of commercial corn-steep solids is reduced and could hinder their acquisition at a large scale. In this sense, partial replacement with peptone A is a beneficial strategy.

Finally, *A. caviae* CHZ306 represents an exciting source of enzymes because it contains different genes encoding chitinases [33]. Moreover, in opposition to the chemical process for chitin degradation, enzymatic hydrolysis is a more sustainable alternative because it does not require the presence of toxic compounds or excessive wastewater generation [56]. The chitin derivatives such as chitosan and chitosaccharides present biological and physicochemical properties interesting for several applications, including biotechnology, biomaterials, food, medicine, cosmetics, wastewater treatments, etc. [56,57]. In this sense, using purified EnCh or ChB in a specific chitin-degradation step and controlling the length of the chitosaccharide would allow the definition of molecules and the evaluation of their biological activity. Additionally, a deep study of the biochemical properties of both enzymes is recommended to obtain a detailed understanding of reaction mechanisms. It is also possible to take advantage of the synthetic capacity of hydrolases in unconventional media (i.e., the synthesis of complex oligosaccharides and polysaccharides).

**Table 3:** Comparative reports of chitinase production by *Aeromonas* species.

Strain	Carbon Source (g.L^−1^)	Nitrogen Source (g.L^−1^)	Time Culture (h)	Chitinase Activity		Ref
				(U.L^−1^)	(U.mg^−1^)	
*Aeromonas caviae* CHZ306	Colloidal chitin (10.0)	Corn-steep solids (1.77) Peptone A (0.51)	12	30 (EnCh) 21.4 (ChB)	nr	This study
*Aeromonas hydrophila* H-2330	Colloidal chitin (5.0)	Polypepton (5.0) Yeast extract (3.0)	24	140	1.7	[58]
*Aeromonas schubertii*	Colloidal chitin (10.0)	Tryptone (1.0) Yeast extract (1.0)	96	155	0.47	[59]
*Aeromonas* sp. GJ-18	Swollen chitin (10.0)	Tryptone (10.0)	120	1,440	14.4	[19]
*Aeromonas* sp. JK1	Colloidal chitin (7.5)	Ammonium sulfate (1.5)	48	9000 *	nr	[31]
*Aeromonas* sp. ZD_05	Colloidal chitin (10.0)	Peptone (7.0)	72	10,000 *	nr	[53]
*Aeromonas punctata* HS6	Colloidal chitin (10.0) Starch (10)	Yeast extract (10.0)	48	82,640	nr	[3]
*Aeromonas hydrophila* HS4	Colloidal chitin (10.0) Starch (10)	Malt extract (10.0)	24–48	86,010	nr	[3]
*Aeromonas* sp. PTCC 1691	Colloidal chitin (7.5)	Ammonium sulfate (1.5)	48	92,000	nr	[35]

* The values were estimated based on the graphics reported in the works; nr = not reported. Ench: endochitinase, and ChB: chitobiosidase.

## 5. Conclusions

Medium composition improvement is relevant in developing a commercial fermentation process affecting microbial growth and product yield. Based on our study, complex nitrogen sources play an essential role in chitinase production. The supplementation of peptone A (M9) and corn-steep solids (M11) as sole nitrogen sources increased EnCh and ChB production by at least 4.1- and 3.6-fold, respectively. In this sense, the organic nitrogen sources, corn-steep solids, and peptone A (at a ratio of 2:1), have been shown to stimulate the cell growth of *A. caviae* CHZ306 and to promote the high synthesis of EnCh (30.1 U.L^−1^) and ChB (21.3 U.L^−1^), in comparison with basal medium, corresponding to more than 5- and 3-fold enhancement, respectively. Further assays should focus on optimizing culture conditions and process implementation in automated bioreactor systems.

## Figures and Tables

**Table 1:** Characteristics of nutrients used for *Aeromonas caviae* CHZ306 growth and chitinase production.

Medium	Nutrient	Manufacturer	Chemical Formula	C (%)	N (%)	Concentration			Final C:N Ratio **
						(g.L^−1^) *	C (g.L^−1^)	N (g.L^−1^)	
M1–M12	Colloid α-chitin	Sigma-Aldrich, Saint Louis, MO, USA	(C_8_H_13_O_5_N)_n_	44.0	0.60	10.0	4.400	0.060	nc
M1	Ammonium sulfate	Sigma-Aldrich, Saint Louis, MO, USA	(NH_4_)_2_SO_4_	0.0	21.2	1.00	0.000	0.212	16.2
M2	Ammonium acetate	Labsynth, Diadema, SP, Brazil	NH_4_CH_3_CO_2_	15.6	18.2	1.17	0.183	0.213	16.8
M3	Ammonium nitrate	Labsynth, Diadema, SP, Brazil	NH_4_NO_3_	0.0	35.0	0.61	0.000	0.214	16.1
M4	Urea	Labsynth, Diadema, SP, Brazil	CH_4_N_2_O	20.0	46.6	0.45	0.090	0.210	16.6
M5	Casaminoacid	BD Biosciences, San Jose, CA, USA	nd	33.3	10.7	1.97	0.656	0.211	18.7
M6	Meat extract	Acumedia, Lansing, MI, USA	nd	41.9	12.5	1.70	0.712	0.213	18.7
M7	Yeast extract	BD Biosciences, San Jose, CA, USA	nd	38.9	10.9	1.94	0.755	0.211	19.0
M8	Peptone Bacto	BD Biosciences, San Jose, CA, USA	nd	43.3	14.9	1.42	0.615	0.212	18.4
M9	Peptone A	Acumedia, Lansing, MI, USA	nd	41.8	13.2	1.61	0.673	0.213	18.6
M10	Peptone G	Acumedia, Lansing, MI, USA	nd	44.3	16.0	1.32	0.585	0.211	18.4
M11	Corn-steep solids	Sigma-Aldrich, Saint Louis, MO, USA	nd	37.6	7.6	2.81	1.057	0.214	19.9
M12	Tryptone	BD Biosciences, San Jose, CA, USA	nd	43.5	12.7	1.67	0.726	0.212	18.8

* The required amount of each nitrogen source (viz. M1–M12) was calculated to achieve approximately 0.2 g.L^−1^ nitrogen supplementation in the mineral-based medium with colloid α-chitin (10 g.L^−1^). C (carbon) and N (nitrogen). ** Final C:N ratio at chitin-mineral medium after individual nutrient supplementation; nd = not determined; nc = not calculated.

**Table 2:** Mixtures of corn-steep solids (M11) and peptone A (M9) in a chitin-containing medium were used for *Aeromonas caviae* CHZ306 cultivation.

Medium (N Ratio) *	Corn-Steep Solids (M11)			Peptone A (M9)			Final C:N Ratio **
	(g.L^−1^)	(C, g.L^−1^)	(N, g.L^−1^)	(g.L^−1^)	(C, g.L^−1^)	(N, g.L^−1^)	
M11–M9 (1:1)	1.33	0.500	0.101	0.76	0.318	0.100	20.0
M11–M9 (1:2)	0.94	0.353	0.071	1.01	0.422	0.133	19.6
M11–M9 (2:1)	1.77	0.665	0.135	0.51	0.213	0.067	26.1

* The required amount of each nitrogen source was calculated to achieve a 0.2 g.L^−1^ nitrogen supplementation in the mineral-based medium with the colloid α-chitin (10 g.L^−1^). C (carbon) and N (nitrogen). ** Final C:N ratio in the chitin-mineral medium after individual nutrient supplementation.

## Data Availability

Not applicable.
